# Case Report: Menstrual cycle–related psychosis in bipolar I disorder, remitting with a combined oral contraceptive

**DOI:** 10.3389/fpsyt.2025.1696891

**Published:** 2025-12-05

**Authors:** Shota Hanyu, Kei Yoshimoto, Nobuo Shimizu

**Affiliations:** Department of Psychiatry, Aino Hanazono Hospital, Osaka, Japan

**Keywords:** menstrual cycle–related psychosis, bipolar disorder, amenorrhea, hyperprolactinemia, estrogen withdrawal, drospirenone–ethinyl estradiol, combined oral contraceptive, case report

## Abstract

Menstrual cycle–related psychosis is a rare condition presenting with recurrent affective and psychotic symptoms temporally linked to the menstrual cycle. We report a case with two uncommon features: paradoxical relapse during antipsychotic-induced amenorrhea with hyperprolactinemia, and sustained remission after adjunctive hormonal stabilization using a combined oral contraceptive (drospirenone–ethinyl estradiol). Although phenomenology overlaps with bipolar disorder, the regular monthly periodicity and frequent premenstrual onset indicate a menstrual cycle–related substrate. Rigorous differentiation from bipolar disorder is therefore essential to avoid misclassification and to guide treatment, including consideration of endocrine modulation when this pattern is evident. We describe a 19-year-old Japanese woman with no psychiatric history who presented with acute mania and psychosis necessitating hospitalization. Despite treatment with antipsychotics and mood stabilizers for a bipolar-spectrum illness, she experienced recurrent monthly relapses tightly linked to menstruation, and prophylaxis could not be achieved. During higher-dose antipsychotic therapy, she developed amenorrhea with marked hyperprolactinemia; however, psychotic relapse occurred even during the amenorrheic period. To stabilize ovarian hormones, a combined oral contraceptive was initiated. Premenstrual exacerbations progressively attenuated and then ceased over four months, permitting simplification to the minimum effective antipsychotic regimen. She remained well for an extended period on maintenance psychiatric pharmacotherapy together with the combined oral contraceptive. This case suggests that menstrual cycle–related psychosis may reflect sensitivity to hypoestrogenism: relapse occurred during antipsychotic-induced amenorrhea, whereas adjunctive treatment with a combined oral contraceptive was associated with sustained remission. Adjustments of antipsychotics and mood stabilizers afforded at most transient benefit and did not prevent relapses or attenuate peak severity; sustained improvement occurred only after continuous drospirenone–ethinyl estradiol. For patients who show clear cycle-linked exacerbations despite antipsychotics and mood stabilizers, endocrine stabilization with a combined oral contraceptive may be considered. Operationally, we coded the case as Bipolar I disorder (DSM-5-TR/ICD-11) based on a syndromal manic episode, while phenotypically it showed a menstrual cycle–related (catamenial) pattern—regular cycle-linked, perimenstrual exacerbations with complete inter-episode remission—distinguishing it from non-catamenial bipolar and other psychotic disorders.

## Introduction

Menstrual cycle–related psychosis is a rare clinical syndrome presenting with acute-onset, cyclical psychotic or manic episodes temporally associated with the menstrual cycle (1, 2). The term is not officially defined in leading classifications such as the Diagnostic and Statistical Manual of Mental Disorders, Fifth Edition (DSM-5) or the International Classification of Diseases, 11th Revision (ICD-11), yet it is used descriptively to denote a substrate associated with the menstrual cycle. Rapid fluctuations in circulating estrogen—particularly perimenstrual declines—are thought to play a central role in its pathophysiology (3, 4). Because the phenomenology often overlaps with bipolar disorder, rigorous differentiation is essential when episodes show regular monthly periodicity and frequent premenstrual onset, both to avoid misclassification and to guide treatment, including the potential role of endocrine modulation (1, 2). Bipolar-disorder literature independently supports cycle-linked vulnerability: a subset of women experience premenstrual exacerbation (PME), and PME is associated with a more symptomatic, relapse-prone course (5). Furthermore, the postpartum period—a natural model of abrupt estrogen withdrawal—carries a high risk of manic or mixed relapse in Bipolar I disorder (6).

Here, we report a 19-year-old woman whose illness course exemplified this pattern, including a paradoxical psychotic relapse during antipsychotic-induced amenorrhea with marked hyperprolactinemia, followed by sustained remission after adjunctive hormonal stabilization with a combined oral contraceptive containing drospirenone–ethinyl estradiol.

## Case description

**Figure 1:**
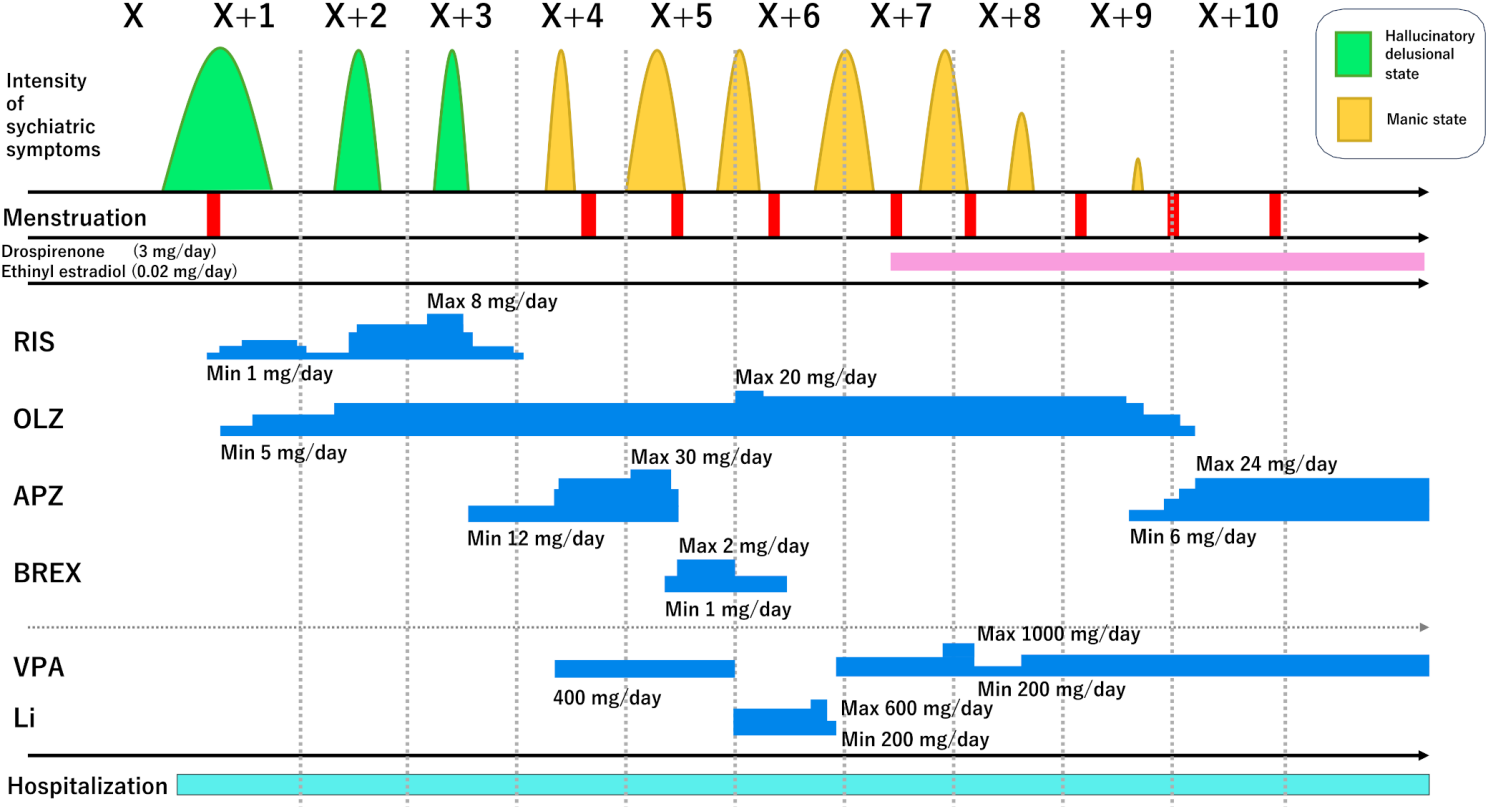
Eleven-month clinical timeline. Psychiatric symptom intensity (green peaks = psychosis; yellow peaks = mania), menstruation (red bars), hospitalization (light blue band), and antipsychotic/mood-stabilizer periods with dose ranges (blue bars; RIS = risperidone, OLZ, olanzapine; APZ, aripiprazole; BREX, brexpiprazole; VPA, valproate; Li, lithium). A pink marker denotes initiation of drospirenone 3 mg/ethinyl estradiol 0.02 mg in Month X + 7.

A 19-year-old Japanese woman with no prior psychiatric history lived with her parents and younger sister after graduating from high school. She was unemployed, reported no alcohol or illicit drug use, and had no prior exposure to psychotropic medications. Her family history included maternal bipolar disorder. The clinical course—encompassing psychiatric symptoms, menstrual cycles, and interventions—is summarized in **Figure 1**. Month-by-month psychotropic dosing and each change’s proximate clinical influence are detailed in **Table 1**.

**Table 1:** Month-by-month psychotropic medication changes and clinical course.

All doses in mg/day	RIS	OLZ	APZ	BREX	VPA	Li	COC	Possible outcome influence
X								No psychotropics; baseline.
X+1	1-3	5-10						Antipsychotics started; effect unclear.
X+2	1-6	10-15						Home-leave worsening; RIS-induced hyperprolactinemia.
X+3	1-8	15	12					Recurrent relapses; etiology unclear.
X+4	0-1	15	12-24		400			APZ + low-dose VPA (± BREX); unstable.
X+5		15-20	12-30	1-2	400			Ongoing fluctuations; no pattern.
X+6		17.5-20		1	0-600	200-600		Li → tremor; stopped; back to VPA; unstable.
X+7		17.5			600-1000		3/0.02	Start COC; acute flares lessened.
X+8		17.5			200-1000		3/0.02	Continued COC; behavior improved; fewer flares.
X+9		10-17.5	6-12		400		3/0.02	Ongoing COC; symptoms settled; less impairment.
X+10		5-10	12-24		400		3/0.02	Post-COC, simplified APs; stability maintained.
X+11			24		400		3/0.02	Remission on COC; minimal additional care.
X+12 - X + 21			24		400		3/0.02	Sustained remission; functional recovery.

All doses are expressed in mg/day; ranges indicate the minimum–maximum daily dose within each month. For COC, “3/0.02” denotes drospirenone 3 mg and ethinyl estradiol 0.02 mg, administered continuously. RIS, risperidone; OLZ, olanzapine; APZ, aripiprazole; BREX, brexpiprazole; VPA, valproate; Li, lithium; COC, combined oral contraceptive.

On Day 28 of Month X, after attending a university open-campus event, she experienced elevated mood and grandiose ideas. By Day 5 of Month X + 1, she had worsened to severe anxiety, persecutory ideation, suspiciousness, and auditory hallucinations (“I’m being watched,” “I can hear children making fun of me”). On Day 6 of Month X + 1, due to escalating psychosis, she was brought to the hospital and admitted the same day for safety.

Evaluation revealed hallucinations, delusional ideation, and loosening of associations (“I am the last person on Earth, and the Earth is spinning backward.”). Physical and neurological examinations revealed no abnormal findings. Head computed tomography (CT) and electroencephalography (EEG) were normal, and blood tests, including metabolic and thyroid function, were within reference ranges. Risperidone 1 mg/day was started and titrated; olanzapine 5 mg/day was added for persistent symptoms. She developed delusions that food and water were “poisoned” and refused intake, requiring brief physical restraint and intravenous fluids. Doses were increased to risperidone 3 mg/day and olanzapine 10 mg/day.

By the end of Month X + 1, about four weeks into hospitalization, her symptoms improved markedly. After two weeks of stable remission, a five-day home leave was initiated on Day 7 of Month X + 2. On Day 3 of leave (Day 9 of Month X + 2), her psychotic symptoms acutely recurred, and she returned to the hospital on Day 11 of Month X + 2, reporting marked distress, crying, shouting, and passivity experiences (“an AI chip was implanted in my head,” thought broadcasting). Medications were maintained at risperidone 6 mg/day and olanzapine 15 mg/day, with gradual restabilization by Day 21 of Month X + 2.

Over the subsequent months, a cyclical pattern of remission and relapse emerged, closely associated with menstruation. In Month X + 4, she exhibited a typical manic episode (overactivity, singing/dancing, expansive mood, grandiosity) that responded to aripiprazole up to 24 mg/day combined with valproate 400 mg/day. The Month X + 4 episode met full DSM-5-TR criteria for mania (distinctly elevated, expansive, or irritable mood with ≥3 associated symptoms and functional impairment requiring hospitalization), supporting coding as Bipolar I disorder, whereas subsequent relapses, including several that again fulfilled syndromal criteria for mania, were tightly cycle-locked exacerbations typically emerging in the perimenstrual period. Brexpiprazole (1–2 mg/day) was added briefly, but the incremental benefit was limited and it was discontinued. Due to recurrent mood fluctuations, valproate was switched to lithium carbonate in Month X + 6; however, disabling tremor at lithium carbonate 200 mg/day developed, prompting a rapid return to valproate with prompt resolution of tremor. Despite optimized psychotropic therapy, relapses continued to align with the menstrual cycle.

In Month X + 7, in light of the strong menstrual cyclicity, a combined oral contraceptive comprising drospirenone 3 mg and ethinyl estradiol 0.02 mg was initiated to stabilize ovarian hormones. As summarized in **Table 1**, brief residual flares persisted immediately after initiation (X + 7) but attenuated over subsequent cycles with simplification of antipsychotic regimens. During that cycle, a brief, mild manic flare occurred, suggesting partial effect. With continued use, impulse control improved by Month X + 8, and psychotic features in the premenstrual period were markedly attenuated. In Month X + 9, adverse effects of olanzapine (finger tremor, cogwheel rigidity) disappeared after discontinuation, and aripiprazole was continued as the sole antipsychotic. By Month X + 10, after approximately four months of continuous drospirenone–ethinyl estradiol in combination with psychotropics, no further relapses were observed. Trial home leaves were uneventful, and she was discharged in Month X + 11. In outpatient follow-up, she remained stable through Month X + 21 on aripiprazole 24 mg/day, valproate 400 mg/day, and a combined oral contraceptive (drospirenone 3 mg/ethinyl estradiol 0.02 mg) daily.

## Discussion and conclusion

This case exhibits a rare cyclical psychosis tightly coupled to the menstrual cycle, consistent with prior reports in which psychotic or manic episodes cluster around menses and respond incompletely to standard psychotropic therapy (1, 2). This report provides two clinically important observations. First, sustained remission followed continuous hormonal stabilization with drospirenone–ethinyl estradiol. Second, relapse occurred during antipsychotic-induced amenorrhea. This indicates that not only abrupt perimenstrual estrogen declines but also residual microfluctuations within an overall hypoestrogenic milieu may precipitate decompensation (3, 4). Taken together, these features support a working diagnosis of menstrual cycle–related psychosis within the bipolar spectrum and highlight an endocrine–psychiatric interface relevant to management.

Under DSM-5-TR and ICD-11 (6A60), the case is coded as Bipolar I disorder because a syndromal manic episode was documented in Month X + 4. Phenotypically, recurrences were temporally locked to the menstrual cycle, typically emerging in the perimenstrual period across cycles, with full inter-episode remission and an endocrinologically plausible framework (antipsychotic-related hyperprolactinemia → GnRH/LH/FSH suppression → relative hypoestrogenism). They showed durable improvement after hormonal stabilization. This profile diverges from primary, non-catamenial bipolar disorder; does not meet schizoaffective disorder (mood episodes did not occupy the majority of total psychotic duration and concurrent mood-psychosis syndromes were not sustained); is atypical for schizophrenia-spectrum disorders; and PMDD does not include psychosis.

Biological plausibility can be organized within a two-axis framework—tonic and phasic. On the tonic axis, sustained hypoestrogenism lowers the relapse threshold by reducing estrogen’s neuromodulatory support for serotonergic and dopaminergic systems and cortical excitability (3, 7). On the phasic axis, rapid perimenstrual estrogen withdrawal and stress-linked HPA activation provide proximal triggers that can tip a vulnerable system into decompensation (1, 3, 4, 8). This tonic-by-phasic model parsimoniously explains the clustering of episodes around menses and other reproductive transitions and aligns with evidence that estrogen modulates dopaminergic and serotonergic systems relevant to antipsychotic response (3, 7).

Recent evidence further substantiates this endocrine framework. In bipolar disorder, premenstrual exacerbation (PME) has been consistently documented and is associated with a more symptomatic, relapse-prone course (5, 10). Moreover, the postpartum period—a model of abrupt estrogen withdrawal—carries a high risk of manic or mixed relapse in Bipolar I disorder, and psychotic features can co-occur in these relapses (6). In parallel, antipsychotic-induced hyperprolactinemia—via suppression of GnRH/LH/FSH and consequent reductions in ovarian estradiol—is consistently linked to menstrual dysfunction and may aggravate psychosis; an umbrella review outlines management options, including aripiprazole augmentation as a prolactin-sparing approach (9).

In this patient, endocrine factors likely contributed directly to relapse generation. Marked hyperprolactinemia under risperidone (105.4 ng/mL) with subsequent amenorrhea supports a chain whereby prolactin suppresses GnRH→LH/FSH, reducing ovarian estradiol/progesterone and establishing a tonic hypoestrogenic state (11). The occurrence of psychotic worsening during amenorrhea aligns with reports that monthly psychosis can occur even without menses (2), implying that relatively small phasic endocrine or neurochemical fluctuations—amplified by stress-responsive HPA–HPG interactions—can trigger relapse once the threshold is lowered (1–3, 6). This observation strengthens the tonic-by-phasic account in a clinically tractable way.

The therapeutic turning point was the initiation of continuous drospirenone–ethinyl estradiol. Conceptually, continuous combined dosing may raise the tonic estrogenic floor while blunting phasic perimenstrual dips, thereby reducing both vulnerability and triggers within the same model (1, 3, 4, 11). After initiation, month-to-month exacerbations attenuated and ceased by Month X + 10, with >10 months of stability thereafter, supporting endocrine stabilization as a pragmatic adjunct in selected patients after risk–benefit discussion.

Strengths of this report include detailed longitudinal characterization, a clear temporal relationship between symptom peaks and menstrual timing, and convergence of the clinical course with a plausible endocrine mechanism. To aid attribution, **Table 1** provides a transparent month-by-month medication timeline aligned with observed symptom changes. Important limitations also warrant emphasis. This is a single case without serial measurements of estradiol, progesterone, LH, or FSH. The absence of contemporaneous hormone assays constrains mechanistic interpretation of the observed temporal coupling. Future work should incorporate prospective, cycle-synchronized serial hormone measurements aligned with standardized symptom ratings (including during antipsychotic-induced amenorrhea and perimenstrual windows) to directly test the hypothesized linkage between ovarian-hormone dynamics and relapse. Spontaneous improvement or placebo effects cannot be fully excluded, and medication adjustments may act as confounders. While such adjustments may have partially contributed to short-term changes, the sustained stabilization coincided with continuous drospirenone–ethinyl estradiol, tempering—but not eliminating—the possibility of confounding. In light of these considerations, the inferences should be viewed as hypothesis-generating rather than definitive.

In clinical practice, it is useful to first consider the contribution of perimenstrual estrogen decline, together with an evaluation of endocrine factors such as antipsychotic-induced hyperprolactinemia and menstrual irregularities. Where appropriate and safe, and after shared decision-making with risk counseling, adjunctive hormonal strategies—aimed at suppressing or stabilizing ovarian hormone fluctuations—may be considered alongside standard psychopharmacologic care.

Taken together, these data integrate prior observations on menstrual cycle–linked psychoses, amenorrhea-period relapses, and estrogen’s receptor-level and HPA-interactive effects into a single tonic-by-phasic framework that generates testable predictions for prospective studies (1–4, 6, 7, 11).

In conclusion, this case emphasizes two points with potential generalizability: sustained remission after hormonal stabilization with drospirenone–ethinyl estradiol, and relapse during antipsychotic-induced amenorrhea. Together, these findings implicate both estrogen withdrawal and residual microfluctuations as candidate triggers. Future prospective studies should delineate phenotypes sensitive to these mechanisms, define endocrine–neurochemical coupling with greater precision, and compare hormonal with non-hormonal approaches to guide personalized treatment.

## Patient perspective

I felt ashamed about things I did during mood highs that didn’t feel like me, and the repeated premenstrual surges disrupted my daily life. Hospitalization, frequent medication changes, and side effects were burdensome, but learning that female hormones likely drove these swings made the pattern understandable. With treatment that took hormones into account alongside my psychiatric care, the surges eased, my sleep and concentration improved, and I’m relieved to feel stable again.

## Data Availability

The original contributions presented in the study are included in the article/**Supplementary Material**, further inquiries can be directed to the corresponding author/s.

## References

[1] Brockington IF . Menstrual psychosis: a bipolar disorder with a link to the hypothalamus. Curr Psychiatry Rep. (2011) 13:193–7. doi: 10.1007/s11920-011-0191-5, PMID: 21424263

[2] Brockington IF . Monthly psychosis during amenorrhoea. Arch Womens Ment Health. (2009) 12:187–8. doi: 10.1007/s00737-009-0054-9, PMID: 19337704

[3] Wharton W Gleason CE Olson SR Carlsson CM Asthana S . Neurobiological underpinnings of the estrogen–mood relationship. Curr Psychiatry Rev. (2012) 8:247–56. doi: 10.2174/157340012800792957, PMID: 23990808 PMC3753111

[4] Mahé V Dumaine A . Oestrogen withdrawal associated psychoses. Acta Psychiatr Scand. (2001) 104:323–31. doi: 10.1034/j.1600-0447.2001.00288.x, PMID: 11722312

[5] Kuehner C Nayman S . Premenstrual exacerbations of mood disorders: findings and knowledge gaps. Curr Psychiatry Rep. (2021) 23:78. doi: 10.1007/s11920-021-01286-0, PMID: 34626258 PMC8502143

[6] Sharma V Wood KN Weaver B Mazmanian D Thomson M . Occurrence of postpartum manic or mixed episodes in women with Bipolar I disorder: a systematic review and meta-analysis. Bipolar Disord. (2024) 26:240–8. doi: 10.1111/bdi.13405, PMID: 38258551

[7] Hwang WJ Lee TY Kim NS Kwon JS . The role of estrogen receptors and their signaling across psychiatric disorders. Int J Mol Sci. (2021) 22:373. doi: 10.3390/ijms22010373, PMID: 33396472 PMC7794990

[8] Oyola MG Handa RJ . Hypothalamic–pituitary–adrenal and hypothalamic–pituitary–gonadal axes: sex differences in regulation of stress responsivity. Stress. (2017) 20:476–94. doi: 10.1080/10253890.2017.1369523, PMID: 28859530 PMC5815295

[9] Jiang Q Li T Zhao L Sun Y Mao Z Xing Y . Treatment of antipsychotic-induced hyperprolactinemia: an umbrella review of systematic reviews and meta-analyses. Front Psychiatry. (2024) 15:1337274. doi: 10.3389/fpsyt.2024.1337274, PMID: 38505795 PMC10948402

[10] Lin J Nunez C Susser L Gershengoren L . Understanding premenstrual exacerbation: navigating the intersection of the menstrual cycle and psychiatric illnesses. Front Psychiatry. (2024) 15:1410813. doi: 10.3389/fpsyt.2024.1410813, PMID: 39176230 PMC11338788

[11] Capozzi A Scambia G Pontecorvi A Lello S . Hyperprolactinemia: pathophysiology and therapeutic approach. Gynecol Endocrinol. (2015) 31:506–10. doi: 10.3109/09513590.2015.1017810, PMID: 26291795

